# Impact of Prematurity and Maternal Bonding on Sensory Processing at 12 Months

**DOI:** 10.3390/children12121581

**Published:** 2025-11-21

**Authors:** Ayse Yildiz, Ramazan Yildiz, Zekiye Basaran, Pelin Atalan Efkere, Rabia Zorlular, Halil Ibrahim Celik, Bulent Elbasan

**Affiliations:** 1 Department of Physical Therapy and Rehabilitation, Faculty of Health Sciences, Erzurum Technical University, 25050 Erzurum, Turkey; 2 Department of Physical Therapy and Rehabilitation, Faculty of Health Sciences, Gazi University, 06560 Ankara, Turkey; zekiyesadbasaran@gmail.com (Z.B.);; 3 Department of Physical Therapy and Rehabilitation, Bor Faculty of Health Sciences, Nigde Omer Halisdemir University, 51240 Niğde, Turkey; rabiaeraslan@ohu.edu.tr; 4 Bilge Cocuk Special Education and Rehabilitation Center, 06800 Ankara, Turkey

**Keywords:** preterm infants, sensory processing, mother-to-infant bonding, mother, relationship

## Abstract

**Highlights:**

**What are the main findings?**

• Preterm infants showed weaker sensory processing skills—particularly in tactile and vestibular domains—compared with term peers.
• Weaker mother-to-infant bonding at 4 months was independently associated with poorer sensory processing outcomes at 12 months.

**What is the implication of the main finding?**

• Early screening and support for mother–infant bonding may help prevent or mitigate sensory processing difficulties in preterm infants.
• Targeted, family-centered interventions focusing on tactile and vestibular stimulation could improve developmental outcomes in this high-risk population.

**Abstract:**

**Background/Objectives**: This study examined the relationship between sensory processing skills, mother–infant bonding, and prematurity in 12-month-old infants. **Methods**: Twenty-two preterm infants with gestational age < 34 weeks and 20 term infants were included in the study. At four months, mothers evaluated their bonding with infants using the Mother-to-Infant Bonding Scale (MIBS). At the age of 12 months, the sensory processing skills of infants were assessed using the Test of Sensory Functions in Infants (TSFI). **Results**: MIBS scores were significantly higher in the preterm group compared to the term group (*p* < 0.01). TSFI total scores and subdomains (tactile deep pressure, adaptive motor function, and vestibular stimulation) were significantly lower in preterm infants (*p* = 0.01, *p* = 0.02, *p* = 0.01, and *p* = 0.03, respectively). Multiple linear regression revealed that each one-point increase in bonding score (weaker bonding) was associated with a −0.65 decrease in the TSFI total score, a −0.61 decrease in tactile deep pressure, and a −0.63 decrease in vestibular stimulation scores, independent of gestational age. **Conclusions**: This study concluded that mother-to-infant bonding is associated with sensory processing skills, especially in the vestibular and tactile domains. Additionally, prematurity was found to be related to sensory processing.

## 1. Introduction

According to the World Health Organization (WHO), infants delivered before completing 37 weeks of gestation are classified as preterm [1]. Despite substantial advances in perinatal and neonatal medicine that have markedly improved survival, these infants continue to face a heightened risk of both short-term complications and long-term developmental challenges [2].

Premature birth is a significant risk factor for neurodevelopmental difficulties affecting sensory processing, emotional regulation, and early mother–infant interactions [3]. Problems with sensory processing skills are seen in 39–52% of preterm infants, and this risk is higher for infants born before the 32nd week of pregnancy [4,5]. Sensory processing skills include recording, modulating, and integrating information from the body and the environment through the nervous system, producing purposeful and goal-directed responses [6]. Sensory processing problems are seen in children who do not adequately process stimuli from various systems, such as tactile, auditory, visual, and vestibular, and exhibit maladaptive responses. Infants born prematurely experience a heightened level of sensory exposure in the Neonatal Intensive Care Unit (NICU), an environment that is considerably more stimulating and stressful than the intrauterine setting. As a result, they face difficulties in oral, tactile, and general sensory processing skills [7]. These sensory processing difficulties may impact their ability to engage in early bonding experiences with their mothers, potentially affecting emotional and social development.

Maternal bonding describes the affective relationship that develops between a mother and her infant during the initial stage following childbirth. It plays a crucial role in caregiving, helping mothers respond to their infants’ needs and creating a secure emotional environment that supports exploration and development. A strong bond contributes to the infant’s emotional, social, and cognitive growth, while disruptions in bonding may lead to long-term developmental challenges [8,9,10]. A weak mother–infant bond causes depression and anxiety in children and also negatively affects the child’s long-term cognitive, socio-emotional, and language development [11]. However, premature birth can disrupt this bonding process due to medical complications, prolonged NICU stays, and reduced physical contact during the critical early days [12]. Additionally, premature infants’ atypical sensory processing may alter their responses to maternal touch, voice, and eye contact, making it difficult for mothers to form a strong emotional bond with their children [13].

Empirical evidence indicates that sensory processing and the mother–infant relationship influence one another in a reciprocal manner. Babies with sensory processing difficulties may be less engaged in social interactions, making it harder for mothers to interpret and respond to cues. Conversely, a strong mother–infant bond may help regulate the baby’s sensory experiences and provide a stabilizing framework for sensory modulation and behavioral responses [13,14]. Although these associations have been theoretically proposed, the interaction between sensory processing and maternal–infant bonding among preterm populations remains insufficiently explored. While the global literature highlights developmental vulnerabilities associated with prematurity, most studies have examined attachment or sensory processing in isolation, emphasizing neurobiological or psychosocial mechanisms [15,16]. The current study addresses this gap by simultaneously assessing both factors in a well-characterized cohort of premature and full-term infants aged 12 months corrected age. This integrative approach contributes to a more comprehensive understanding of how prematurity, early attachment, and sensory development interact; this evidence can inform early intervention and parent–infant support strategies in neonatal follow-up programs worldwide.

## 2. Materials and Methods

### 2.1. Participants

The Ethics Committee of Gazi University (E-77082166) approved the study using relevant ethical standards. The Declaration of Helsinki was used to conduct the study.

A total of forty-two mother–infant pairs participated in the study. The control group included twenty term infants whose gestational ages ranged between 37 and 41 weeks. The preterm group comprised twenty-two infants born before 34 weeks of gestation and weighing under 1500 g at birth. Informed consent for participation was obtained from all mothers on behalf of both themselves and their infants. Infants suspected of chromosomal defects, hydrocephalus, cerebral palsy, congenital heart disease, metabolic or neuromuscular disease, and those born to mothers unable to speak Turkish were excluded from the study in both groups.

### 2.2. Procedures

All infants included in the study were evaluated at 4 and 12 months of age by two therapists with an average of 10 years of experience in pediatric rehabilitation. All assessments were conducted through direct observation by trained pediatric physiotherapists. The therapist who assessed the infants at 12 months of age was blind to the results of the 4-month assessment and the perinatal history of the infants. Mothers completed the MIBS independently when their infants were four months of corrected age, and therapists could explain the items as necessary. Sensory processing skills were assessed using the TSFI when the infants reached the corrected age of one year. The TSFI was administered in a quiet clinical setting, with infants actively participating. The assessments were conducted in real time, and item scoring was done immediately based on the infant’s responses.

### 2.3. Measures

The Mother-to-Infant Bonding Scale (MIBS) was employed to assess the emotional attachment between mothers and their infants. This instrument, recognized for its validity and reliability, identifies maternal challenges in developing a positive relationship with the newborn from the first postpartum week through the fourth month. The MIBS consists of 8 different statements, each consisting of an emotion: loving, resentful, disappointed, neutral, joyful, dislike, protective, and aggressive. The total score ranges from 0 to 24, where higher values reflect greater bonding difficulties [17]. The Turkish adaptation of the MIBS, validated by Karakulak et al., demonstrated acceptable psychometric properties (Cronbach’s α = 0.71), confirming its suitability for use among postpartum women in Turkey [18].

The Test of Sensory Functions in Infants (TSFI) evaluates sensory processing and responses in infants ages 4 to 18 months [19]. This evaluation comprises subtests including tactile deep pressure reactivity, adaptive motor behavior, visual–tactile integration, oculomotor coordination, and vestibular responses. The 24-item test yields a composite score ranging from 0 to 49, with elevated scores indicating greater sensory processing difficulties. The Turkish version of the scale was used in the study. The Turkish adaptation of the TSFI demonstrated good reliability and validity (Cronbach’s alpha = 0.875), supporting its applicability in evaluating sensory functions in infants aged 4 to 18 months in Turkey [20].

### 2.4. Data Analysis

Statistical analyses were performed utilizing the Statistical Package for the Social Sciences (SPSS) for Windows, version 25.0 (SPSS Inc., Chicago, IL, USA). Visual methods, such as histograms, and analytical techniques, such as the Shapiro–Wilk test, determined the normal data distribution. Data with a normal distribution were summarized as mean values accompanied by standard deviations, whereas non-normally distributed variables were presented as medians with interquartile ranges. Between-group comparisons for continuous variables following a normal distribution were performed using independent samples *t*-tests. When the assumption of normality, verified through the Shapiro–Wilk test, was not met, the Mann–Whitney U test was applied as a nonparametric alternative. Categorical variables were analyzed using chi-square tests to determine group differences.

The initial linear regression analysis in the total sample indicated a confirmed relationship between TSFI scores and bonding. In the simple linear regression model, when the association was significant (*p* < 0.05), adjustments for potential confounding factors, identified through descriptive analyses, were included in the subsequent multiple linear regression analysis. Variables with evident clinical relevance or those displaying significance in a univariate test with a *p*-value below 0.1 were incorporated into the regression models. The multiple linear regression models assessed and addressed outliers and adherence to multicollinearity assumptions. A *p*-value of <0.05 was considered statistically significant.

The current study was completed with a total of 42 infants. Sample size and post hoc power analysis were performed with G*Power (version 3.1.9, Universität Düsseldorf, Düsseldorf, Germany). The post hoc power analysis was performed after the study, and it was found to be 92.4%, 93.6%, 90%, and 80.8% for the regression models with a statistical significance of α = 0.05, number of predictors = 2, and sample size = 42.

## 3. Results

The study was completed with 42 infants (22 preterm and 20 term controls) (Table 1). There was no difference between the groups concerning the gender distribution of infants, maternal education level, or maternal age. The mean age of mothers in the preterm group was 30.94 (SD = 3.90) years, while it was 28.79 (SD = 5.75) years in the term group, with no statistically significant difference between groups (*p* = 0.21). Regarding education level, most mothers in both groups had completed 12 years of education or more, with no significant difference between the groups (*p* = 0.78). There was a statistically significant difference between the groups regarding the MIBS score (*p* < 0.01). The preterm infants demonstrated lower performance across the TSFI total score and its subdomains related to tactile deep pressure reactivity, adaptive motor responses, and vestibular stimulation compared to their term counterparts.

The simple linear regression models reveal a significant correlation between bonding and various TSFI scores, including vestibular stimulation reactivity (t = −2.09, *p* = 0.04), reactivity to deep tactile pressure (t = −3.72, *p* < 0.01), adaptive motor responses (t = −2.40, *p* = 0.02), and the overall total (t = −3.54, *p* < 0.01). Associations remain statistically significant after including gestational age as a confounding factor in multiple linear regressions, except for adaptive motor responses (Table 2).

A substantial correlation was observed between bonding and a significant decrease in TSFI scores, manifesting as a decline of −0.65 points in the total score, −0.61 points in reactivity to deep tactile pressure, and −0.63 points in vestibular stimulation reactivity. In this context, a low MIBS score indicates a mother–infant solid bond, which causes the correlation to be negative. Concurrently, a correlation existed between gestational age and a reduction in the corresponding TSFI scores (Table 2).

Within the preterm group, there was no significant correlation between the total TSFI score and gestational age (t = −1.14; *p* = 0.27) or bonding (t = −1.79; *p* = 0.09). The reduction in gestational age and the increase in bonding score did not significantly decrease the total TSFI score. Similarly, in the control (term-born) group, no significant association was found between bonding and total TSFI score (t = −0.28; *p* = 0.78).

## 4. Discussion

This study showed that mother–infant bonding is linked to sensory processing, while prematurity is also related to sensory processing at 12 months. While these findings are consistent with international evidence, they fill an important gap in the literature where there is limited research examining these developmental dynamics in premature infants.

The need for bonding begins at birth, with the baby needing its parents and surroundings. Studies examining the bonding process between mothers and babies in preterm children have reported that premature birth negatively affects the bonding process. Preterm delivery and the ensuing separation between mother and infant can abruptly disrupt the establishment of maternal–infant bonding, thereby postponing and diminishing the mother’s caregiving responses [21,22]. Previous studies have also indicated that mothers of infants born at earlier gestational ages may experience more pronounced difficulties in forming a secure bond [23]. Preterm birth rates have gradually increased worldwide, and the neonatal intensive care experience often includes prolonged hospital stays, restricted parental contact, and heightened emotional distress for mothers [24,25]. Studies have shown that mothers with infants in NICUs report higher levels of anxiety and depression, which can negatively influence bonding [26]. Despite the well-documented difficulties associated with prematurity, limited research has explored its long-term influence on sensory maturation and mother–infant attachment. Consistent with previous findings, the present study demonstrated that maternal bonding tends to be more vulnerable among infants born preterm. This phenomenon is hypothesized to be associated with the premature group staying in the NICU. Mutlu et al. revealed that mothers whose infants were in the NICU had weak emotional bonds with their babies [24]. Studies have also shown that long periods of infants staying in intensive care hurt the quality of mother–infant interaction [26]. The admission of a newborn infant to the NICU is typically an unanticipated occurrence for families. During pregnancy, parents may not contemplate the likelihood of their newborn infant requiring hospitalization. The admission of an infant to the NICU is a profoundly stressful experience for parents. This unexpected situation often evokes intense emotions, including shock, denial, anger, and guilt [27]. Familial challenges and the infant’s medical condition can harm the mother–infant relationship [28].

Sensory processing plays a central role in shaping infants’ overall development by supporting the integration of motor, cognitive, emotional, and social systems. Efficient processing of sensory input allows infants to interpret environmental cues, regulate arousal, and engage in purposeful interactions that promote learning and exploration [29]. Conversely, delayed or atypical sensory processing may disrupt these developmental cascades. Infants who exhibit hypersensitivity or hyposensitivity to sensory input often experience difficulties with motor coordination, attention, emotion regulation, and social responsiveness [30]. Such early disruptions can lead to cumulative challenges, including delayed language acquisition, behavioral dysregulation, and reduced participation in age-appropriate activities during early childhood [31]. Given the central role of sensory processing in regulating infants’ physiological and emotional states, alterations in sensory experiences are also likely to influence early relational patterns, particularly the processes underlying mother–infant bonding.

The admission of preterm infants to the NICU during a period of rapid brain development, coupled with exposure to stimuli that are incongruent with their sensory needs, has a detrimental impact on their sensory skills [32]. Cabral et al. observed changes in the response to deep tactile pressure and vestibular stimulation in premature infants with a corrected age of 4 to 6 months [33]. Bart et al. observed that sensory modulation challenges manifest in various aspects for one-year-old preterm children, encompassing reactions to deep pressure, adaptive motor behavior, visual-tactile integration, and vestibular reactions [34]. A study examining preterm infants at a corrected age of 4–12 months found that the most frequently impacted responses were tactile deep pressure and vestibular stimulation [35]. In line with the literature, this study concluded that premature infants at 12 months of corrected age exhibited poor performance in response to tactile deep pressure, adaptive motor function, and response to vestibular stimulation. These findings support the hypothesis that premature infants are more susceptible to abnormal sensory processing than their term counterparts.

Understanding how sensory processing relates to bonding is critical, as infants who exhibit atypical sensory patterns often struggle to interpret and respond to environmental input, which in turn may influence the quality of their bonding behaviors. Current multidisciplinary research suggests that the prevalence and patterns of sensory processing disorders impact parent–child relationships [36]. However, no studies examine the relationship between attachment and sensory processing skills in preterm children. The present study revealed a relationship between bonding, deep tactile pressure, and vestibular stimulation subscales of sensory processing skills. It is not surprising that the deep tactile sensation affects sensory processing. Among the sensory systems, tactile perception is the first to develop during intrauterine life and serves as a cornerstone for the emergence of early bonding behaviors [37]. The maternal-fetal bond is reinforced postnatally through the mother and infant’s interactions, including eye contact, olfactory stimulation, and tactile contact [38]. After delivery, bonding is encouraged by a skin-to-skin connection between the baby and the mother [39]. Through physical and tactile interactions, mothers help modulate their infants’ physiological stability and emotional regulation. Touch is vital to caregiving, fulfilling basic needs, and facilitating regulatory processes crucial for developing bonding. Consequently, a logical correlation exists between touch scores and bonding.

Despite observing a significant association between bonding and sensory processing in the overall sample, no statistically significant relationship was found between MIBS scores and TSFI scores within the preterm group alone. Several potential factors may explain this outcome. First, the relatively small number of participants in the preterm group might have reduced the statistical power required to identify significant associations. Second, the variability in individual neonatal experiences—such as differences in NICU environments, length of stay, parental visitation patterns, and medical complications—may have introduced confounding effects that diluted the relationship. Additionally, sensory processing development in preterm infants is influenced by a complex interplay of biological, environmental, and caregiving factors; thus, bonding alone may not be a strong enough predictor when isolated from these broader influences. Future studies should consider larger, more stratified preterm samples and include additional variables such as NICU exposure and quality of early interactions to clarify this relationship better.

Existing literature presents inconsistent findings regarding the determinants of sensory processing difficulties in preterm populations. Pekçetin et al. observed that infants with sensory challenges were characterized by lower gestational age and birth weight [40]. Similarly, Adams et al. identified both gestational age and birth weight as significant predictors of sensory processing symptoms among preschool-aged preterm children [41]. In contrast, Crozier et al. reported that gestational age was not related to sensory processing outcomes, whereas prolonged hospitalization in the NICU was associated with increased sensory processing differences [42]. Machado et al. examined sensory processing skills in 12-month-old preterm children and found no impact of gestational age or birth weight on these skills. Similarly, Celik et al. found no significant relationship between premature infants’ gestational age, birth weight, and the TSFI total score [43]. Our study found that bonding level and gestational age did not affect sensory processing skills in the preterm group. This outcome is explained by the fact that sensory processing skills in premature infants are not only related to immaturity at birth. Indeed, they are also influenced by various biological and environmental factors [40].

The study indicated a correlation between premature birth and mother–infant bonding and sensory processing. However, it should be noted that other factors may also affect the infant’s sensory development. As a result, these findings should be interpreted with caution. Furthermore, maternal postnatal bonding occurs within broader family dynamics, where siblings and other caregivers also play a role.

Limitations: A limitation of this study is that it focuses exclusively on the mother-child bond. Future research should emphasize the simultaneous role of both parents’ bonding processes from a family-centered perspective. One more limitation of the study is the relatively small sample size. While post hoc power analysis demonstrated adequate statistical power (between 80% and 93% across regression models), the limited number of participants may limit the generalizability of the findings. This limitation is largely due to the stringent inclusion criteria, which ensured a homogeneous sample of premature infants free of confounding neurological or metabolic conditions. However, the current results provide valuable exploratory data that can guide future large-scale, multicenter studies investigating the relationship between maternal bonding and sensory processing in premature infants across diverse cultural and clinical contexts. Also, the small number of preterm participants and the inability to classify according to gestational age (e.g., early and late preterm) may have masked the relationship between bonding and sensory processing skills. Another limitation of the study is the lack of information about the period between the 4-month bonding assessment and the 12-month sensory processing evaluation. Numerous environmental and developmental factors during this critical time may have affected bonding and sensory development. Future studies should include longitudinal tracking with intermediate assessments to better understand the evolving dynamics between early bonding and later sensory outcomes.

## 5. Conclusions

In conclusion, this study highlights the association between mother–infant bonding and sensory processing, particularly in the tactile and vestibular domains, and the impact of prematurity on these outcomes. While emphasizing the importance of early bonding, future interventions should adopt a comprehensive family-centered approach that includes mothers, fathers, and other primary caregivers. Father-infant bonding and the support provided by extended family members or caregivers may play a protective role in the infant’s sensory and emotional development, particularly in prematurity. Expanding the focus beyond maternal bonding can contribute to more holistic and effective early developmental support strategies for preterm infants. Beyond its relevance to pediatric physiotherapy and developmental assessment, the findings of this study have broader implications across multiple disciplines. Understanding how early bonding and sensory processing interact can inform the design of interdisciplinary early intervention programs that integrate physical, emotional, and social components of infant care. For instance, developmental psychologists and occupational therapists may use these insights to design sensory-enriched environments that promote parent–infant co-regulation, while neonatologists and public health professionals can incorporate bonding-oriented sensory stimulation protocols into neonatal care practices. Moreover, these results contribute to the growing field of neurodevelopmental science by emphasizing the need to examine early sensory–emotional interactions as foundational mechanisms underlying later cognitive, behavioral, and relational outcomes. Integrating such perspectives may ultimately improve developmental trajectories and family well-being on a broader societal level.

## Figures and Tables

**Table 1:** Descriptive statistics for infants.

	Preterm (N = 22)	Control (N = 20)	*p*
	Mean (SD) Median [IQ Range]	Mean (SD) Median [IQ Range]	
Age (months)-N (%)	12.26 (0.26)	12.10 (0.20)	0.29 ^a^
Infant gestational age (weeks)	29 [27.2–33]	39 [37.5–40]	**<0.01 ^b^**
Infant birth weight (g)	1200 [1000–1500]	3315 [3001.2–3513.7]	**<0.01 ^b^**
Days in the neonatal unit	51.47 (8.46)	8.07 (10.36)	**<0.01 ^a^**
Male sex-N (%)	12 (54.5%)	8 (40%)	0.35 ^c^
Maternal age (years)	30.94 (3.90)	28.79 (5.75)	0.21 ^a^
Maternal education years			0.78 ^c^
Primary school	2	1	
High school	9	10	
University	11	9	
MIBS scores	4.5 [3–6]	0.5 [0–1.75]	**<0.01 ^b^**
TSFI scores Total	35.5 [31.25–42.5]	45 [41–47]	**0.01 ^b^**
Reactivity to tactile deep pressure	8 [6–10]	10 [9.25–10]	**0.02 ^b^**
Adaptive motor function	11 [9–14]	14.5 [13–15]	**0.01 ^b^**
Visual-tactile integration	9 [7.5–10]	10 [9–10]	0.64 ^b^
Ocular-motor control	2 [2–2]	2 [2–2]	0.95 ^b^
Reactivity to vestibular stimulation	7.5 [5.75–10]	10 [9–10]	**0.03 ^b^**

Number of participants (N); percentage (%); standard deviation (SD); interquartile (IQ); Mother-to-Infant Bonding Scale (MIBS); Test of Sensory Functions in Infants (TSFI). *p* < 0.05 highlighted in bold. ^a^: *t*-Test, ^b^: Mann–Whitney test, ^c^: Chi-square test.

**Table 2:** Multiple linear regression model within the total sample predicting sensory processing.

Outcomes	Explanatory Variables	B	*β*	SE	*p*	r	R^2^_adj_
Total TSFI score	Constant	57.63		19.87			
	Bonding	−0.65	−0.13	1.01	**0.02**	0.59	0.262
	Gestational age	−0.29	−0.07	0.51	**0.01**		
Reactivity to deep tactile pressure score	Constant	12.04		5.28			
	Bonding	−0.61	−0.12	0.27	**0.03**	0.56	0.272
	Gestational age	−0.19	−0.05	0.14	**0.02**		
Adaptive motor responses score	Constant	20.81		8.32			
	Bonding	−0.58	−0.12	0.42	0.56	0.57	0.246
	Gestational age	−0.26	−0.07	0.21	0.38		
Reactivity to vestibular stimulation score	Constant	16.20		5.94			
	Bonding	−0.63	−0.13	0.30	**0.04**	0.37	0.202
	Gestational age	−0.18	−0.05	0.15	**0.02**		

Test of Sensory Functions in Infants (TSFI); unstandardized partial regression coefficient (B); standardized coefficient (β); standard error (SE); effect size (r). The constant is the expected mean value of the TSFI scores in the control group. *p* < 0.05 highlighted in bold.

## Data Availability

The data presented in this study are available on request from the corresponding author due to ethical reasons.

## Abbreviations

The following abbreviations are used in this manuscript:

WHO	World Health Organization
NICU	Neonatal Intensive Care Unit
MIBS	Mother-to-Infant Bonding Scale
TSFI	Test of Sensory Functions in Infants
